# Efficient plant regeneration through direct shoot organogenesis and two-step rooting in *Eucommia ulmoides* Oliver

**DOI:** 10.3389/fpls.2024.1444878

**Published:** 2024-09-20

**Authors:** Dacheng Wang, Pengfei Su, Yameng Gao, Xue Chen, Wenjie Kan, Jinyan Hou, Lifang Wu

**Affiliations:** ^1^ The Center for Ion Beam Bioengineering & Green Agriculture, Hefei Institutes of Physical Science, Chinese Academy of Sciences, Hefei, China; ^2^ School of Life Science, University of Science and Technology of China, Hefei, Anhui, China; ^3^ Taihe Experimental Station, Hefei Institutes of Physical Science, Chinese Academy of Sciences, Fuyang, Anhui, China

**Keywords:** adventitious root, axillary bud sprouting, *E. ulmoides*, shoot elongation, two-step rooting

## Abstract

*Eucommia ulmoides* Oliver (*E. ulmoides* Oliver), a multipurpose woody plant, holds great economic significance due to its expansive medicinal, food and industrial applications. The rapid advancement of *E. ulmoides* in various fields has resulted in the inadequacy of existing breeding methods to meet its growth and annual production demands. Consequently, there is an urgent need for innovative propagation strategies. This study introduces an optimized micropropagation protocol for *E. ulmoides*, facilitating direct shoot organogenesis from nodal segments with axillary buds. We systematically examined the impact of basal medium composition, plant growth regulators, photosynthetic photon flux density, and sucrose concentration on bud sprouting. Employing cuttings with axillary buds as propagation material, we achieved a shortened cultivation period of merely 4 weeks for bud elongation and proliferation, marking a substantial enhancement in propagation efficiency. Notably, the Driver Kuniyuki Walnut medium, supplemented with 20.0 g L^−1^ sucrose and 2.0 mg L^−1^ trans-zeatin, induced shoots sprouting with a 100% success rate and an average length of 5.18 cm per nodal segment, equating to a great bud propagation rate of approximately 500%. Furthermore, a light source with an intensity of 80 μmol m^−2^ s^−1^ was shown the most economical choice. To address the primary challenge of inducing roots in regenerated plants, we employed a refined two-step rooting technique. This method yielded the optimal rooting frequency of 93.02%, producing an average of 5.90 adventitious roots per plantlet, each with an average length of 2.77 cm. The micropropagation program developed in this work will be the cornerstone for the preservation of the germplasm of *E. ulmoides* and its long-term use in medicinal and industrial applications.

## Introduction

*Eucommia ulmoides* Oliver (*E. ulmoides* Oliver), commonly referred to as “Du-zhong”, a perennial deciduous tree, is a living fossil plant and the only species both in its genus and in its family (Wang et al., 2003). The species is known to thrive under diverse soil and climatic conditions, and is prevalent in twenty-eight provinces of China (Wang C. Y. et al., 2019). *E. ulmoides* is a valuable tree species in China, serving as a critical industrial raw material and a component of herbal medicine. In addition, as one of the most important woody oils, *E. ulmoides* helps to preserve soil and water, while simultaneously enhancing the aesthetic appeal of the landscape (Bao et al., 2024).

*E. ulmoides* as a traditional herbal has been widely used for more than two thousand years, and has been listed in “China Pharmacopoeia” (2020 Edition) (Yao et al., 2012). Currently, many studies have shown that the bark can be used as medicine as leaves and male flowers, and it contains a variety of bioactive components, such as lignans, megastigmane glycosides, iridoids, phenolics, and flavonoids. Pharmacological activities of these bioactive compounds include neuroprotective anti-tumor, anti-oxidative, anti-inflammatory and anti-hypertensive effects (Sun et al., 2022; Sih et al., 1976; Qu et al., 2006; Ding et al., 2022; Wang Y. S. et al., 2019). Owing to its considerable medicinal value, it has been subjected to excessive exploitation in its natural habitat. The IUCN Red List of Threatened Species conducted its most recent evaluation of *E. ulmoides* in 2018, classifying the species as Vulnerable according to criterion D1 (https://www.iucn.org/). The cultivation of *E. ulmoides* is expanding in China due to the rising demand and increasing value associated with the species.

The industrial value of the plants primarily lies in *Eucommia ulmoides* rubber (EUR), which holds an equally significant position alongside its medicinal value. *E. ulmoides* is a species endemic to China and a high-quality gum species with the greatest potential for development in the world, with only a small number of introductions and cultivations abroad (Du, 2010). The supply of natural rubber resources is closely related to the lifeblood of the national economy. In addition, *E. ulmoides* is increasingly being recognized for its potential in the fields of tea, wine and beverage, and cosmetic industries. Given the extensive application of *E. ulmoides* both domestically and internationally, the market potential is immense. Consequently, the advancement of the *E. ulmoides* industry necessitates a consistent availability of superior quality plantlets to ensure sustainable productivity. Nonetheless, the absence of a reliable and consistent plant tissue culture system has impeded the widespread cultivation of *E. ulmoides* and the utilization of transgenic technology. Thus, it is essential to establish a robust and dependable regeneration system for *E. ulmoides*. In production, *E. ulmoides* is mainly propagated through traditional sowing methods (Du, 2010). However, the seeds are encased in a coat and samara with EUR, resulting in a low natural germination rate and asynchronous germination time (Deng et al., 2021). Similarly, the induction of adventitious roots (AR) poses a significant challenge for the large-scale propagation of *E. ulmoides* through cuttings, primarily due to inherent material constraints and seasonal limitations. *E. ulmoides* is a woody plant that is difficult to cultivate. Currently, the efficient micropropagation of *E. ulmoides* remains problematic due to the inherent specificity of the woody material, resulting in low propagation efficiency and challenging induction of AR. Previous studies have successfully demonstrated the potential for plant regeneration from various plant tissues, such as hypocotyls (Chen et al., 2007), juvenile axillary buds (Li et al., 2019), leaves (Wang, 2016), and petioles (Zhu et al., 2001). For instance, previous studies have successfully induced adventitious shoots from 2-week-old hypocotyls (Chen et al., 2008). However, it is important to note that the hypocotyls used in this study are derived from daughter material and may not accurately reflect the genetic background of the original superior variety (Sidorova et al., 2017). While Li et al. (2004) developed a regeneration protocol in which young branches were used as explants, however, the rooting rate only reached a maximum of 57.5%. Effective *E. ulmoides* regeneration systems are now the main obstacle preventing its widespread use.

The efficient regeneration system for *E. ulmoides* has not yet been fully established, which continues to pose a significant challenge in this field of research. Current research primarily focuses on improving the production of adventitious buds to enhance multiplication efficiency. Prior to this, extensive investigations have already been undertaken concerning the induction of adventitious buds in *E. ulmoides*. However, it is exceptionally challenging to induce a significant number of adventitious buds across the different tissues and organs of *E. ulmoides* (Chen et al., 2008; Zhang S. W. et al., 2024; Tang and Jin, 2010). Regardless of the induction method employed, the resulting adventitious buds are often weak and in poor growth condition (Ran et al., 2022; Wu et al., 2024). Furthermore, before adventitious buds can be utilized for propagation or rooting, they must first undergo a phase of robust growth, which is divided into two distinct steps. This process not only consumes more time but also often fails to achieve the ideal state for plants.

Tissue culture-mediated micropropagation technique can be inexpensively and widely used. Nevertheless, the success of the multiplication process and the survival rate of regenerated, acclimated plants determine how cost-effective the method are. Before acclimation, it is crucial to make sure the plants have a robust root system. In this investigation, we tackled the challenge of poor root development in *E. ulmoides* by employing a novel two-step rooting propagation method. Our primary focus is on enhancing the propagation efficiency of *E. ulmoides*. In the present study, we developed a direct organ regeneration approach, which uses juvenile stem segments to directly promote axillary bud sprouting and elongation under *in vitro* conditions, in order to overcome the difficulties of regeneration and low proliferation efficiency. As new shoots are directly developed from superior stem segments to form axillary buds, this process preserves the positive traits of the donor plants and doesn’t result in genetic diversity. This approach is of paramount theoretical and practical significance.

## Materials and methods

### Plant material and primary explants preparation and sterilization

**Figure 1:**
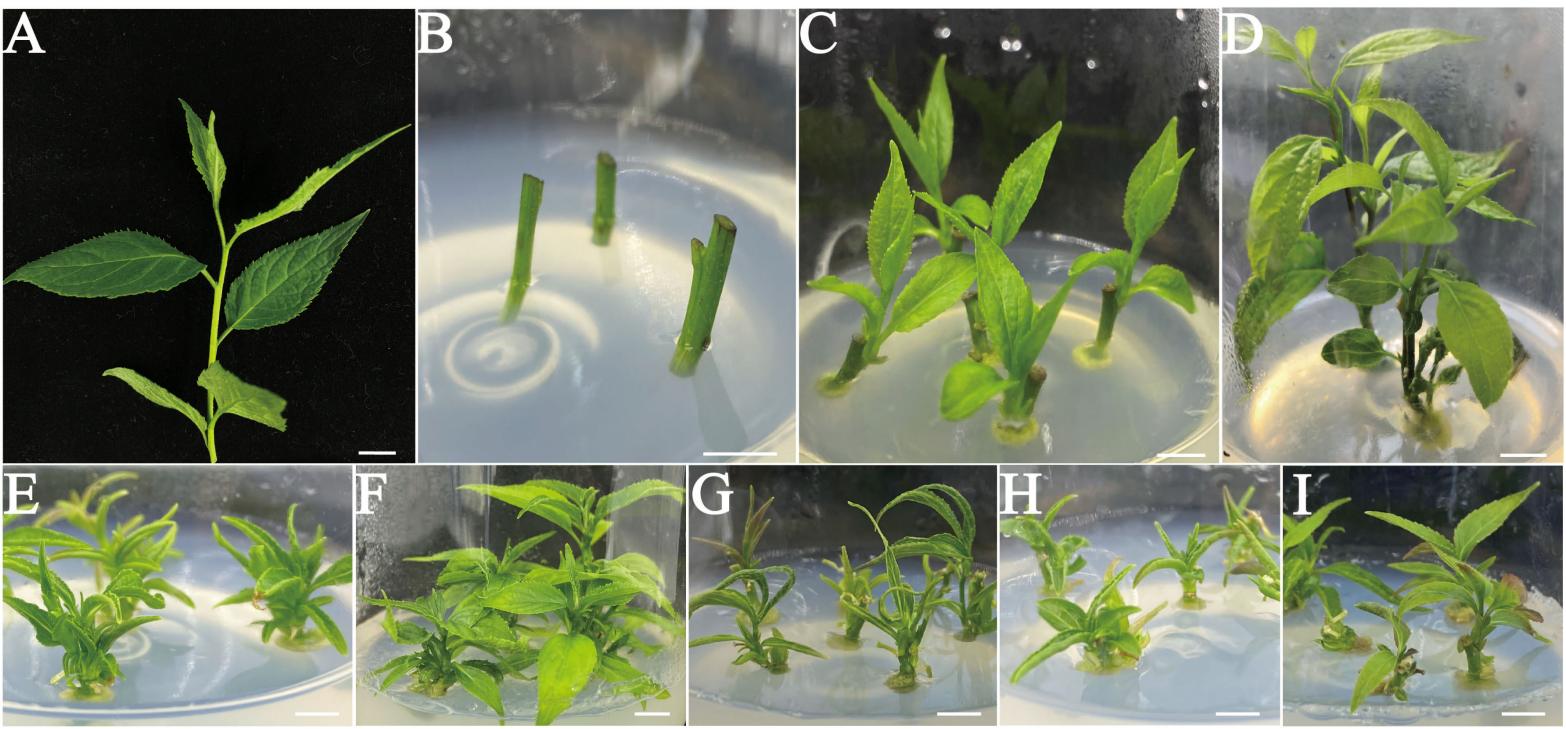
*In vitro* shoot initiation from nodal segments of *E. ulmoides.* **(A)** Fresh shoot segments were collected from a mature and healthy *E. ulmoides* tree in April. **(B)** The surface-sterilized single node shoot stems with axillary buds in the sprouting MS medium supplemented with 0.5 mg L^–1^ 6-BA. **(C)** Shoot development from surface-sterilized single node stem segments with axillary buds on the MS basal medium supplemented with 0.5 mg L^–1^ 6-BA for 2 weeks. **(D)** Shoot development and elongation of the surface-sterilized explants cultivated on the MS basal medium fortified with 0.5 mg L^–1^ 6-BA for 4 weeks. **(E-I)** Single node shoot development from axillary buds on the different basal media fortified with 0.5 mg L^–1^ 6-BA. **(E)** B5. **(F)** DKW. **(G)** N6. **(H)** MS. **(I)** WPM. (Bars = 1.0 cm).

To minimize the issues related to endophytes and browning during disinfection, this study selected fresh shoots from April as explants. The donor material collected from a 9-year-old *E. ulmoides* tree was obtained from the Hefei Institutes Physical Sciences, Chinese Academy of Sciences in Anhui Province (N 31° 49′, E117° 13′). According to the methodology employed in our prior patent (Wu et al., 2023), fresh branch sections (**Figure 1A**) with axillary buds were carefully prepared by removing the leaves and cutting them into stem segments measuring approximately 5 cm in length. Subsequently, the segments were washed for 20 min. Next, the nodal stems were immersed in a solution of 75% (v/v) ethanol for 45 s. After that, the stem segments were disinfected with a 0.1% (w/v) HgCl_2_ solution for 3 min. After rinsing with sterile distilled water 6 times, residue disinfectant was thoroughly removed from the explants. To prepare the stem segment for further experimentation, the necrotic parts were removed from both ends. Then, the stem segments were trimmed to approximately 1 cm in length, ensuring the presence of at least one axillary bud to serve as the initial explant. Explants were cultured vertically (**Figure 1B**) in Murashige and Skoog (MS) medium (Murashige and Skoog, 1962) supplemented with 0.5 mg L^−1^ N6-Benzyladenine (6-BA), 30.0 g L^−1^ sucrose, and 6.8 g L^−1^ agar. The culture media were adjusted to a standardized pH of 5.8, and the conditions for cultivation were regulated at a temperature of 25 ± 2°C. Additionally, the photoperiod was set to 14 h, with a photosynthetic photon flux density (PPFD) of 50 μmol m^−2^ s^−1^. The disinfected stem segments, obtained from explants, were cultured over a 4-week period to prepare for the subsequent phase of the experiment.

### Effects of the medium type on shoot sprouting

This experiment aimed to investigate the impact of various culture media types on the sprouting and growth of isolated stem segments after a 4-week culture period. The new shoots were cut and cultured onto five different basal culture media: Gamborg’s B-5 Basal (B5) Medium (Gamborg et al., 1968), MS medium, Woody Plant (WPM) Medium (Lloyd and Mccown, 1980), N6 medium (Chu et al., 1975), and the Driver Kuniyuki Walnut (DKW) medium (Driver and Kuniyuki, 1984). Each medium contained 0.5 mg L^−1^ of 6-BA, 30.0 g L^−1^ sucrose, and 6.8 g L^−1^ agar. Stem segments were cultured for 4 weeks to generate the inoculated material. To ensure an ample supply of materials for the “Shoot sprouting and proliferation” experiment, we initiated subsequent experiments following five consecutive subculturing generations on an optimized medium.

### Shoot sprouting and proliferation

In this study, we utilized regenerated shoots that had undergone five subculturing cycles as our experimental material. We sectioned these shoots (approximately 1 cm in length), meticulously preserving a single node with an axillary bud to facilitate bud sprouting and development. The segments were subsequently cultured vertically in DKW medium, supplemented with 0.5, 1.0 or 2.0 mg L^−1^ of N6-furfuryl adenine (Kn), 6-BA, or thidiazuron (TDZ); or 0.5, 1.0, 2.0 or 3.0 mg L^−1^ of trans-zeatin (tZ), respectively. Plant growth regulators (PGRs), including 6-BA, Kn, and α-naphthaleneacetic acid (NAA), were purchased from Sigma-Aldrich, USA. In contrast, tZ and TDZ used in the present study were sourced from Shanghai Sangon Bioengineering, China.

In the control group (C), no PGRs were employed. Additionally, an investigation was carried out to study the impacts of varying PPFD (20, 50, 80, 110, and 140 μmol m^−2^ s^−1^) and sucrose concentrations (10, 15, 20, 25, and 30 g L^-1^) on the sprouting of shoots *in vitro* using 30-day-old nodal segments, aiming to stimulate the growth of elongated shoots. The DKW medium, supplemented with 2.0 mg L^−1^ of tZ, was employed for the sprouting of axillary buds. The responses to shoot sprouting were evaluated after a cultivation period of 4 weeks.

### Rooting *in vitro*

In this experiment, a two-step rooting protocol was employed with the aim of increasing the AR induction rate and reducing the duration required for root development. The shoots, which had grown well and uniformly to a height of 3-4 cm, were trimmed at the base to a length of approximately 0.5 cm. Stem cuttings were transferred to 1/2 DKW medium containing 1.0, 2.0, 3.0 or 5.0 mg L^−1^ NAA for 1, 3, 5, 7 or 9 days, after which they were transferred to 1/2 DKW medium without NAA. The treatment groups that received NAA at 1.0 mg L^−1^ for 1, 3, 5, 7 or 9 days were labelled 1dN1, 3dN1, 5dN1, 7dN1 and 9dN1, respectively, and the other NAA treatment groups were named accordingly. At least 30 elongated buds were used per treatment. The entire rooting treatment lasted a total of 4 weeks, during which the percentage of rooting, number of ARs and root length were measured.

### Root development process and histological observation

To gain a deeper understanding of the process of AR formation induced by NAA, stem cuttings (3-4 cm in height) of *E. ulmoides* were divided into three groups: one group was initially placed on 1/2 DKW medium supplemented with 3.0 mg L^−1^ NAA and cultured for 7 days. Subsequently, they were transferred to 1/2 DKW medium devoid of NAA, identified as 7dN3. The remaining cuttings were randomly allocated into two groups and transferred to 1/2 DKW medium containing 0.0 or 3.0 mg L^−1^ NAA, denoted as CK and N3, respectively. We chose particular times during the 7dN3 group-induced rooting (0, 3, 5, 7, 9, and 11 days). Photographs were captured under the SZX10 stereomicroscope (Olympus, Japan) on days 0, 3, 5, 7, 9 and 11 of treatment. Furthermore, paraffin-embedded tissue sections were meticulously prepared from the collected samples and subjected to histological examination using a BX53 microscope (Olympus, Japan). At the same time, samples for section observation were collected on the 11th day of the N3 group.

### Acclimatization of *in vitro* regenerated plantlets

To enhance adaptation to the new environment, plantlets with robust AR were carefully extracted from the containers. They were then carefully washed under running tap water to remove any agar that could have adhered to the roots. After that, the plants were moved onto clear seedling trays with nutritional matrix that had been previously watered and sanitized. The nutrient matrix used in our previous study had a ratio of 1:1:1 of perlite, vermiculite, and soil (Hou et al., 2020a). At last, the plants were transferred to a greenhouse maintained at a temperature of 25 ± 2°C and had plastic coverings on to maintain an 80% relative humidity for the first 2 weeks. Subsequently, the lid was removed, and the regenerated plantlets were allowed to further develop in the greenhouse for two months before transplantation into the field.

### Statistical analysis and visualization

This study used a completely randomized design (CRD) to administer the treatments in three independent experiments, each experiment with at least 30 explants. The data was analyzed using GraphPad Prism 8 (GraphPad Software, USA) software, and figures were assembled in Adobe Illustrator 2021 (Adobe Systems, USA). Data was analyzed statistically using one-way analysis of variance (ANOVA) with Duncan’s test using SPSS 26. At *P* < 0.05, there was a statistical difference between the means.

## Results and discussion

### Initiation of shooting

This experiment opted for juvenile shoots with axillary buds as explants (**Figure 1A**). It is commonly known that the best materials for micropropagation are axillary buds or terminal buds of juvenile stem segments. Two weeks later, the axillary or terminal buds successfully sprouted and developed into delicate green shoots (**Figure 1C**). Throughout the subsequent 4 weeks period, the nascent shoots exhibited progressive elongation, increasing both in length and size of stem segments and leaves. (**Figure 1D**). The current research on the development of *E. ulmoides* axillary buds was still quite limited. Studies has revealed that endogenous levels of IAA tend to increase during the bud development process, as evidenced by the transcriptomic analysis of three distinct regions within the axillary bud. This suggests that an elevated IAA content is conducive to bud sprouting (Zhang et al., 2023).

The choice of explants is pivotal in dictating the quality of plant regeneration, serving as a decisive element in gauging a plant’s regenerative potential. *E. ulmoides*, a dioecious perennial woody plant, presents challenges in terms of regeneration and efficient genetic transformation. Currently, the primary available system for *in vitro* regeneration relies on propagation of shoots from seedling organs, as the seeds are genetically different from the parents due to cross-fertilization. Moreover, other tissues from mature *E. ulmoides* trees have proven to be recalcitrant to *in vitro* regeneration.

### Effect of the basal media type on shoot initiation

Indeed, all basal media tested were able to induce axillary bud sprouting at 100% (**Table 1**). However, in MS medium supplemented with 0.5 mg L^−1^ 6-BA, the axillary buds’ capacity to elongate gradually declined, and growing stage leaves gradually start to show signs of yellowing (**Figure 1H**). Consistent findings were also noted in B5, N6, and WPM media (**Figures 1E, G, I**). In terms of axillary shoot length (3.11 ± 0.10 cm) and growth status, explants cultivated on the DKW medium outperformed the others (**Figure 1F**).

**Table 1:** Effect of different types basal media, all supplemented with 0.5 mg L^−1^ 6-BA, on shoot development from single nodes with axillary bud of *E. ulmoides* after 4 weeks of cultured.

Type of Medium	Axillary bud sprouting rate (%)	Mean shoot length (cm)
B5	100.00 ± 0.00^a^	1.48 ± 0.19^bc^
DKW	100.00 ± 0.00^a^	3.11 ± 0.10^a^
MS	100.00 ± 0.00^a^	1.75 ± 0.03^b^
N6	100.00 ± 0.00^a^	1.25 ± 0.17^c^
WPM	100.00 ± 0.00^a^	1.76 ± 0.02^b^

Values are mean ± SD of three independent experiments, each experiment contained at least 30 explants. Values followed by different letters in the same column represent significance at *P* < 0.05 by Duncan’s test.

For plants to grow and develop in tissue culture, it is essential to choose an appropriate media. The most commonly recommended tissue culture medium is the MS basal medium. Nevertheless, media specifically formulated for the cultivation of woody plants, exemplified by DKW or WPM, should place a premium on fostering robust growth and optimal development (Nezami-Alanagh et al., 2018). Furthermore, B5 and N6 culture media were also examined. In our observations, *E. ulmoides* exhibits robust growth on DKW medium. However, the exploration of alternative media by researchers is quite limited, as the majority have predominantly utilized the MS medium for their studies (Chen et al., 2007; Zhang S. et al., 2024; Wu et al., 2024). This underscores the necessary for a broader investigation into the effects of various media on the sprouting and development of *E. ulmoides*.

### Shoot sprouting and elongation

**Figure 2:**
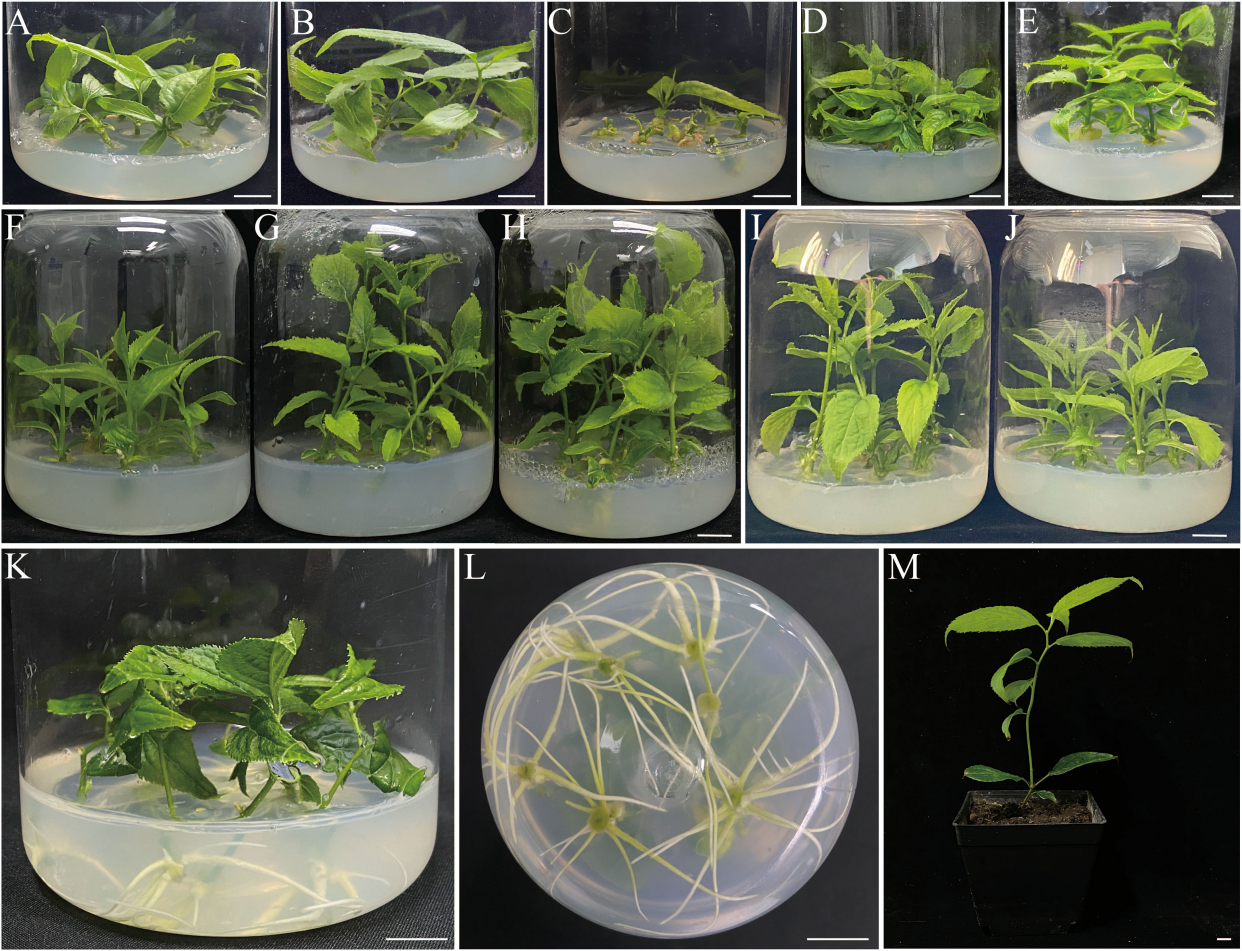
*In vitro* direct shoot organogenesis and AR induction from the single-node explants of *E. ulmoides.* **(A-E)** The effect of different exogenous cytokinins on shoot sprouting from nodal segments with axillary bud of *E. ulmoides* after 4 weeks of cultured on DKW media supplemented with **(A)** C 0.0 mg L^–1^. **(B)** Kn 1.0 mg L^–1^. **(C)** TDZ 0.5 mg L^–1^. **(D)** 6-BA 0.5 mg L^–1^. **(E)** tZ 2.0 mg L^–1^. **(F-H)** The effect of PPFD on nodal segments with axillary buds elongation of *E. ulmoides* after 4 weeks of treatment at **(F)** PPFD 20 μmol m^–2^ s^–1^. **(G)** PPFD 80 μmol m^–2^ s^–1^. **(H)** PPFD 140 μmol m^–2^ s^–1^. **(I-J)** The effect of sucrose content on axillary bud elongation after 4 weeks of cultivation on DKW medium supplemented with sucrose at **(I)** 20 g L^–1^. **(J)** 10 g L^–1^. **(K, L)** *In vitro* rooting of the elongated shoots of the 7dN3 treat group for 4 weeks of culture. **(M)** *In vitro* rooted plantlets acclimatized in the greenhouse for 2 months. (Bars = 1.0 cm).

Firstly, we experimented to determine the optimal conditions for inducing buds sprouting. To accomplish this, we tested different types of cytokinins. The results demonstrate the crucial role of exogenously applied cytokinins in bud sprouting (**Table 2**). After 4 weeks of observation, the explants cultured in Kn medium showed incomplete sprouting of the axillary buds with low shoots’ height. The outcomes aligned with C (**Figure 2A**). The distinction was that Kn promoted bud elongation more strongly (**Figure 2B**). Explants cultured into TDZ-containing media exhibited notable inhibition of bud primordia sprouting and elongation, as well as the production of bigger basal callus than other treatments (**Figure 2C**). This phenomenon may be attributed to TDZ having dual functions of cytokinin and auxin, which are essential for controlling endogenous growth regulators (Singh et al., 2003).In contrast, the sprouting and elongation of pre-existing axillary bud primordia were notably improved when the explants were cultivated on media with a lower concentration of 6-BA, specifically below 2.0 mg L^−1^. However, this approach does not directly achieve the goal of elongation (**Table 2**; **Figure 2D**). Analyzing four types of cytokinins, the results showed that tZ was more advantageous for the elongation of *E. ulmoides* shoots (**Table 2**). Numerous other plants species, such as Persimmons (Liu et al., 2023), *Vaccinium arboreum* (Li et al., 2021), *Toona sinensis Roem* (Hou et al., 2022), have also demonstrated that tZ was superior to other cytokinins in inducing shoots sprouting. When assessing economic factors alongside shoots growth status, we propose that the optimal cytokinin concentration for promoting shoot sprouting and elongation is 2.0 mg L^−1^ of tZ, specifically within the concentration range of 2.0 or 3.0 mg L^−1^ (**Table 2**; **Figure 2E**). The average shoot length was recorded as 4.33 ± 0.01 cm on DKW medium. The regenerated elongating buds should be segmented for further propagation. In this study, we adopted single axillary bud leafless segments as propagation materials. Approximately every 1 cm segment was defined as a propagation material, and a growing bud could be divided into 4 to 6 segments after four weeks of cultivation (**Supplementary Figure S1**). In addition, each segment could enter the next round of propagation in just 4 weeks. This indicates that the propagation efficiency would be four to six times (400%-600%) for per round (cycle) of propagation, and it will increase with each propagation cycle. Moreover, this approach not only ensures a better growth state but also outperforms the traditional method of inducing adventitious buds sprouting followed by elongation, showcasing its superior efficacy in *E. ulmoides* propagation.

**Table 2:** Effect of different exogenous cytokinins on shoot development from single nodes with axillary bud of *E. ulmoides* after 4 weeks cultured on DKW medium.

PGRs (mg L^−1^)		Axillary bud sprouting rate (%)	Mean shoot length (cm)
C Kn TDZ 6-BA tZ	0 0.5 1.0 2.0 0.5 1.0 2.0 0.5 1.0 2.0 0.5 1.0 2.0 3.0	100.00 ± 0.00^a^ 94.44 ± 0.07^a^ 97.22 ± 0.06^a^ 94.44 ± 0.07^a^ 86.79 ± 0.03^bc^ 82.16 ± 0.05^c^ 84.72 ± 0.02^b^ 100.00 ± 0.00^a^ 100.00 ± 0.00^a^ 100.00 ± 0.00^a^ 100.00 ± 0.00^a^ 100.00 ± 0.00^a^ 100.00 ± 0.00^a^ 100.00 ± 0.00^a^	1.72 ± 0.09^f^ 2.37 ± 0.07^e^ 2.51 ± 0.13^de^ 2.42 ± 0.08^de^ 1.39 ± 0.06^g^ 1.39 ± 0.02^g^ 1.30 ± 0.15^g^ 3.19 ± 0.05^b^ 3.12 ± 0.03^b^ 2.85 ± 0.05^c^ 2.61 ± 0.16^d^ 3.23 ± 0.20^b^ 4.33 ± 0.01^a^ 4.50 ± 0.18^a^

Values are mean ± SD of three independent experiments, each experiment contained at least 30 explants. Values followed by different letters in the same column represent significance at *P* < 0.05 by Duncan’s test.

The precise regulation of axillary bud growth is crucial for the development of plant morphology that are suited to their specific environmental conditions (Leyser, 2009). It has been suggested that enhanced cytokinins production and subsequently increased cytokinins availability to buds are necessary for the liberation of buds from auxin-mediated apical dominance after decapitation (Müller et al., 2015). Therefore, in a variety of woody plant species, exogenous cytokinins have been employed to stimulate the development of multiple shoots from the apical portions of stems. It is known that auxin inhibits the production of cytokinins and that cytokinins promote the development of buds. Nevertheless, different plant species have varying preferences for specific types and concentrations of cytokinins that facilitate the growth and multiplication of shoots.

Current studies on the regeneration system of *E. ulmoides* are relatively scarce, with most studies depicting buds that are not thriving and exhibit yellowing leaves (Wu et al., 2024; Zhang S. W. et al., 2024), suggesting challenges in achieving robust growth. The predominant reliance on 6-BA or its combination with NAA in studies (Chen et al., 2007; Li et al., 2019; Huang et al., 2024) coupled with a lack of exploration into other formulations, may be hindering the achievement of higher regeneration rates. More importantly, previous studies have attempted to induce the production of multiple buds, which typically requires a two-step process: first inducing the buds, followed by their elongation. This leads to an extension of the timeline. In contrast, our approach can simultaneously achieve bud sprouting and elongation, significantly reducing the time required.

### Effect of PPFD on axillary bud sprouting and elongation

**Figure 3:**
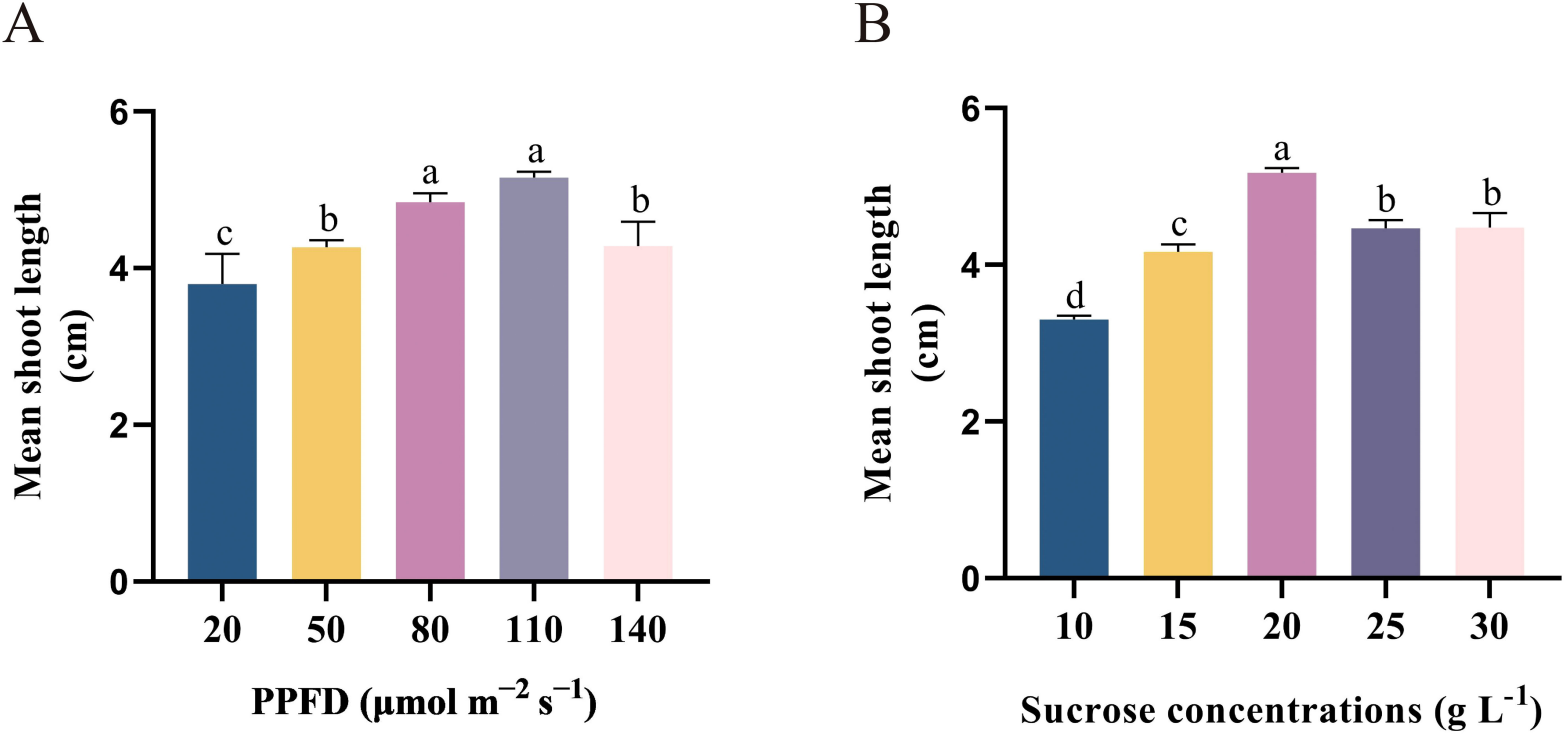
Effect of **(A)** PPFD and **(B)** sucrose concentration on axillary bud elongation from single-node segments isolated from *in vitro*-grown plants and cultivated on DKW medium supplemented with 2.0 mg L^–1^ tZ for four weeks. Bars represent standard errors. Different letters indicate significant differences between different treatments (p < 0.05) by Duncan’s test.

To examine the effects of PPFD on the initiation and elongation of shoots, shoots were cultivated in varying PPFD (20, 50, 80, 110, and 140 μmol m^−2^ s^−1^) on DKW medium that supplemented with 2.0 mg L^−1^ tZ. The results demonstrated the influence of PPFD on shoot initiation and elongation efficiency. After four weeks of cultivation, shoot height gradually increased with the PPFD (ranging from 20 to 110 μmol m^−2^ s^−1^) increased (**Figure 3A**). The highest response in shoot elongation was observed at a PPFDof 110 μmol m^−2^ s^−1^ (5.16 cm). In fact, the length of the shoots was short when the PPFD was below 80 μmol m^−2^ s^−1^ (**Figure 3A**). In addition, at lower PPFD, the leaves and stems displayed a more tender green color and exhibited minimal shoot length (3.80 cm), as illustrated in **Figure 2F**. Plantlets exhibited a greater degree of lignification and maturity at PPFD 140 μmol m^−2^ s^−1^, and a notable inhibition of shoot growth was noted (**Figures 3A**, **2H**). This study suggests that an optimal PPFD of 80 μmol m^−2^ s^−1^ should be chosen to enhance plant growth. As the picture shows (**Figure 2G**), there was a great improvement in shoot length (4.84 cm). At the same time, using this PPFD results in a reduction in the need for lamps compared to a PPFD of 110 μmol m^−2^ s^−1^, thereby lowering energy consumption. These findings align with Zi Wang’s report, which attributed the growth slowdown in *Arabidopsis* under high PPFD causes the development of reactive oxygen species (ROS) as a result of stress (Wang et al., 2022).

Light plays a pivotal role in the *in vitro* cultivation of plants, greatly affecting their developmental progress and growth rate. The intensity of light is crucial for the growth, morphogenesis, production, and physiological responses of *E. ulmoides* plants. To ensure the robust growth and the production of health-enhancing phytochemicals in plants, it is crucial to supply them with optimal light intensity that aligns with their specific needs. Under high PPFD, ginseng leaves experience photoinhibition, resulting in leaf bleaching and premature senescence, on the other hand, in field cultivation under low PPFD, root production is diminished (Lee et al., 2022). In the study of micropropagation of *Ananas comosus* L. Merr, an optimal PPFD of 80 μmol m^−2^ s^−1^ was found to be significantly more effective in promoting the proliferation of secondary shoots compared to a higher PPFD of 100 μmol m^−2^ s^−1^ (Cavallaro et al., 2023).

### Effect of sucrose content on axillary buds sprouting and elongation

The current study aimed to investigate the impact of sucrose concentration on axillary buds sprouting and elongation of *E. ulmoides* in order to minimize production costs. The findings demonstrated that, up to the sucrose concentration reached 20 g L^−1^, the sprouting shoot response was proportionate to the amount of sucrose in the medium (**Figure 3B**). The medium enriched with 2.0 mg L^−1^ tZ and 20.0 g L^−1^ sucrose showed a mean shoot length of 5.18 cm (**Figure 2I**). Our findings also indicate that when exposed to higher concentrations of sucrose (30 g L^−1^), the growth of *E. ulmoides* plantlets is hindered and the leaves show signs of yellowing. This suggests that excessive sucrose can induce cell senescence, aligning with numerous previous studies (Vanrensburg and Vcelar, 1989; Sunitibala et al., 1998; Zheng et al., 2022). Insufficient sucrose content similarly leads to a deficiency in carbon source and energy supply, impairing plant growth and resulting in stunted plant growth (**Figure 2J**).

Sucrose plays a crucial role as a carbon source and energy provider for plants. Carbon serves as the fundamental source of energy required for cell growth. However, in the case of *in vitro* culture, most plant cell cultures are cultivated in a heterotrophic environment, and the plants are unable to acquire energy through natural photosynthesis (Hann et al., 2023). Therefore, to fulfill the nutritional and energy requirements of plants, it is necessary to introduce carbon sources that can be directly utilized by them into the culture medium. Sucrose, which is abundant and vital in plants, can serve as an immediate energy source for explants. In plant tissue culture, a commonly employed sucrose concentration for bud sprouting is 30 g L^-1^ (Hou et al., 2020b; Savitikadi et al., 2020; Zheng et al., 2022). However, the impact of sucrose concentration has been scarcely investigated at elongation stage.

### *In vitro* rhizogenesis and acclimatization

**Figure 4:**
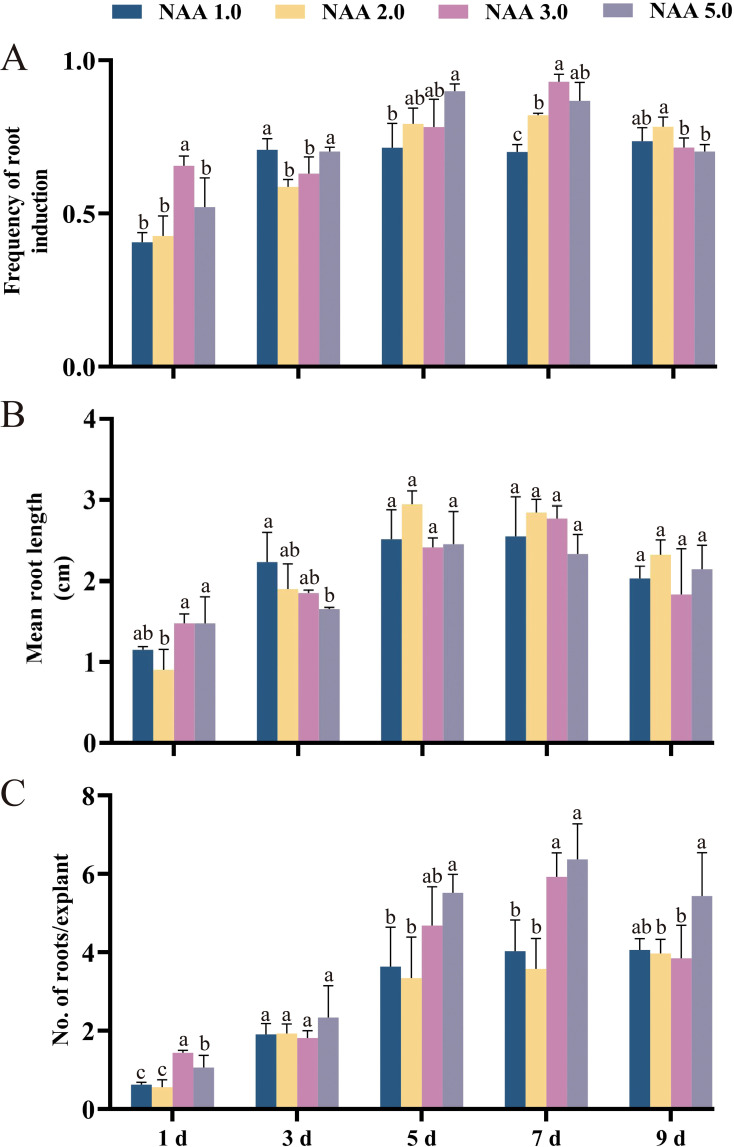
The effect of preculture of elongated shoots of *E. ulmoides* on 1/2 DKW medium supplemented with 1.0, 2.0, 3.0 or 5.0 mg L^–1^ NAA for 0, 1, 3, 5, 7 or 9 days on *in vitro* rooting. **(A)** Frequency of root induction. **(B)** Mean root length. **(C)** Number of roots per explant. Bars represent standard errors. Different letters indicate significant differences between different treatments (p < 0.05) by Duncan’s test.

During this study, we applied a two-step rooting method for the first time to induce AR in *E. ulmoides*. The two-step rooting method involves pre-culturing in a medium containing auxin for a period of time, followed by transferring to a hormone-free medium for further culturing. Although the two-step rooting method has been used in certain woody species, it is still relatively under-researched. Studies have shown the main reason was that auxin promoted root induction and initiation but inhibited root elongation (Bai et al., 2020). The findings demonstrated that the amount of auxin NAA present and the duration of pretreatment significantly influence the formation of AR in *E. ulmoides* (**Figure 4**). As the pre-treatment duration for each NAA concentration increased, there was a corresponding increase in the frequency of AR induction, the average number of AR, and the average length of AR before 7 days of culture (**Figure 4**). However, after a pre-culturing time of 7 days, these indicators started to decrease (**Figure 4**). Notably, after 9 days of NAA pre-treatment, all measures of AR induction showed a significant decrease (**Figure 4**). The group 7dN5 exhibited the highest average count of AR compared to the other groups, with a maximum of 6.4 roots. However, the average length of its AR was only 2.36 cm. Based on the experiment results, it is evident that the group treated with 7dN3 exhibited the highest rate of AR induction (93.02%). Additionally, this group displayed an average of 5.90 ARs, with an average length of 2.77 cm. Consequently, we consider this group to be the most favorable experimental combination.

We also observed that elongating buds treated with NAA at various concentrations for one day had an average of less than two ARs. As the duration of pre-treatment increased from one to seven days, the average number of AR gradually increased. However, after 9 days of pre-treatment, the average number of AR began to decrease, displayed similar patterns to the average root length and induction rate. Therefore, determined that the 7dN3 group was the optimal condition for inducing root formation in *E. ulmoides* elongating shoots, as the 7dN3 group showed well-developed roots after 4 weeks of cultivation (**Figures 2K, L**).

The regenerated plantlets have the exact same form as the mother plant and are quite healthy after two months (**Figure 2M**). With a 92.5% survival rate, the test-tube plantlets were effectively tamed.

Sufficient branch roots and high survivorship of environment-adapted plantlets are necessary for the effective regeneration of plantlets. It is commonly acknowledged that auxin, an essential plant hormone, is critical to the formation of AR. Auxin has been widely recognized as the principal phytohormone that controls and regulates root growth and development. The mechanism by which auxin regulates rooting has been extensively investigated (Ding et al., 2021; Seo et al., 2021; Wei et al., 2019). Different plants species may exhibit varying responses to different auxins (NAA, IBA, and IAA). Previous studies have meticulously examined the physiological indexes, transcriptomes, and metabolomes of two *E. ulmoides* varieties that exhibit varying propensities for AR formation. These comparative analyses have unveiled substantial disparities in the concentrations of IAA, abscisic acid, jasmonic acid, and salicylic acid across the two varieties. The findings have pinpointed the pivotal factors and principal pathways that are crucial for the adventitious rooting process in *E. ulmoides* (Du et al., 2024). The conventional method for inducing AR *in vitro* involves a one-step method. Specifically, adventitious buds are cultivated in a medium containing auxin until they develop into plantlets (Hou et al., 2020b; Heydari and Chamani, 2022; Li et al., 2022; Huang et al., 2022).

*E. ulmoides*, a tree species known for its challenging rooting process, presents a significant technical hurdle that restricts its commercial micropropagation. Currently, there is limited scientific literature concerning the development of AR in *E. ulmoides*. In the existing research literature, the majority of researchers employ a one-step rooting method and continue the culture in a medium containing auxin until the plant reaches full development (Chen et al., 2007; Wang, 2016; Chen et al., 2008). Nonetheless, some researchers have utilized rooting powder ABT (Aminobenzotriazole) to treat the stem base, immersing it briefly before inserting it into a hormone-free medium to induce root development (Zhu et al., 2001). However, the AR induction rate associated with these methods typically remained at around 50%, considerably limiting the development of the *E. ulmoides* regeneration system. Previous studies have employed a two-step rooting approach to effectively address the challenges associated with low induction rates and a limited number of ARs for certain hard-to-root woody plants. A two-step procedure developed for *in vitro* rooting of tea microshoots. The plantlets were incubated on 1/3 MS medium supplemented with 25.0 µM NAA for 10 days, and then transferred to auxin-free 1/3 MS medium, resulting in a remarkable 100% rooting success rate (Bag and Palni, 2010). Similarly, during the micropropagation process of apple, IBA pretreatment for 3, 5, 7, and 27 days achieved a 100% rooting rate (Bai et al., 2020).

The prevalent one-step rooting methods in *E. ulmoides* research have been found to be less efficient. Li et al. (2004) reported a 57.5% rooting rate with the White medium as the most effective, and Zhu et al. (2001) achieved the highest rate of 78.6% using ABT treatment for 3-5 s followed by placement in 1/4 MS medium. These efficiencies are restrictive for the industry’s development. Moreover, the scarcity of detailed regeneration protocols and visual documentation in existing literature impedes the evaluation of the robustness of the regeneration systems.

When it comes to the ability of adventitious shoots to develop into complete plants, the presence and condition of the root system are crucial. For woody plants that are challenging to root, the two-stage rooting technique is provided to be an efficient way to increase both the rate and quality of root development. This method shortened the rooting time and greatly increased the AR induction rate. Based on our current understanding, the two-step rooting method has not yet been employed in *E. ulmoides* tissue culture, and there is limited research on this topic as well. To enhance the survival rate of transplanted regenerated plants and minimize costs, we have introduced the two-step rooting method in regenerated *E. ulmoides* plants, marking the first instance of its application.

### Root development and morphological anatomy

Based on our above experiments, significant advantages in root development were observed in the treatment group subjected to 7dN3. The process of AR induction was subsequently observed in both the group without the addition of auxin NAA (referred to as CK) and the N3 group. However, in the N3 group, roots were generated with a significant amount of callus formation observed, along with yellowing and senescence of leaves (**Supplementary Figure S2**). These adverse effects significantly impacted the survival rate. In contrast, the control group (CK) did not exhibit any root or callus tissue development. In this study, we also observed that the 7dN3 group initiated the production of AR on the 11th day of the experiment, whereas the stem segments in the N3 group only exhibited further expansion. In contrast, the stem base of the control group (CK) showed no signs of root development (**Supplementary Figure S2**). This study proposes that the involvement of auxin NAA in the rooting of *E. ulmoides* is primarily confined to its initial stimulation phase. However, during the subsequent stage of root elongation, high concentrations of NAA may potentially act as an inhibitory factor.

The formation of AR is a complex biological process influenced by various internal and external factors. Anatomically, AR formation begins with the induction phase, during which the plant detects the stimulus but does not undergo significant cell division. Concurrently, target cells reprogram to generate new meristematic cells. Subsequently, the induction phase is succeeded by the formation of AR primordia (initiation phase). In the final phase, the primordia differentiate into roots, with the differentiated vascular bundles connecting to the vascular cylinder of the stem, ultimately resulting in the emergence of roots (AR emergence phase) (Lei et al., 2018). Therefore, the process of AR formation can be categorized into three stages: induction, initiation, and emergence (Li et al., 2018; Mao et al., 2020). According to the observations conducted in this study, the development process of AR in *E. ulmoides* follows the aforementioned rules.

**Figure 5:**
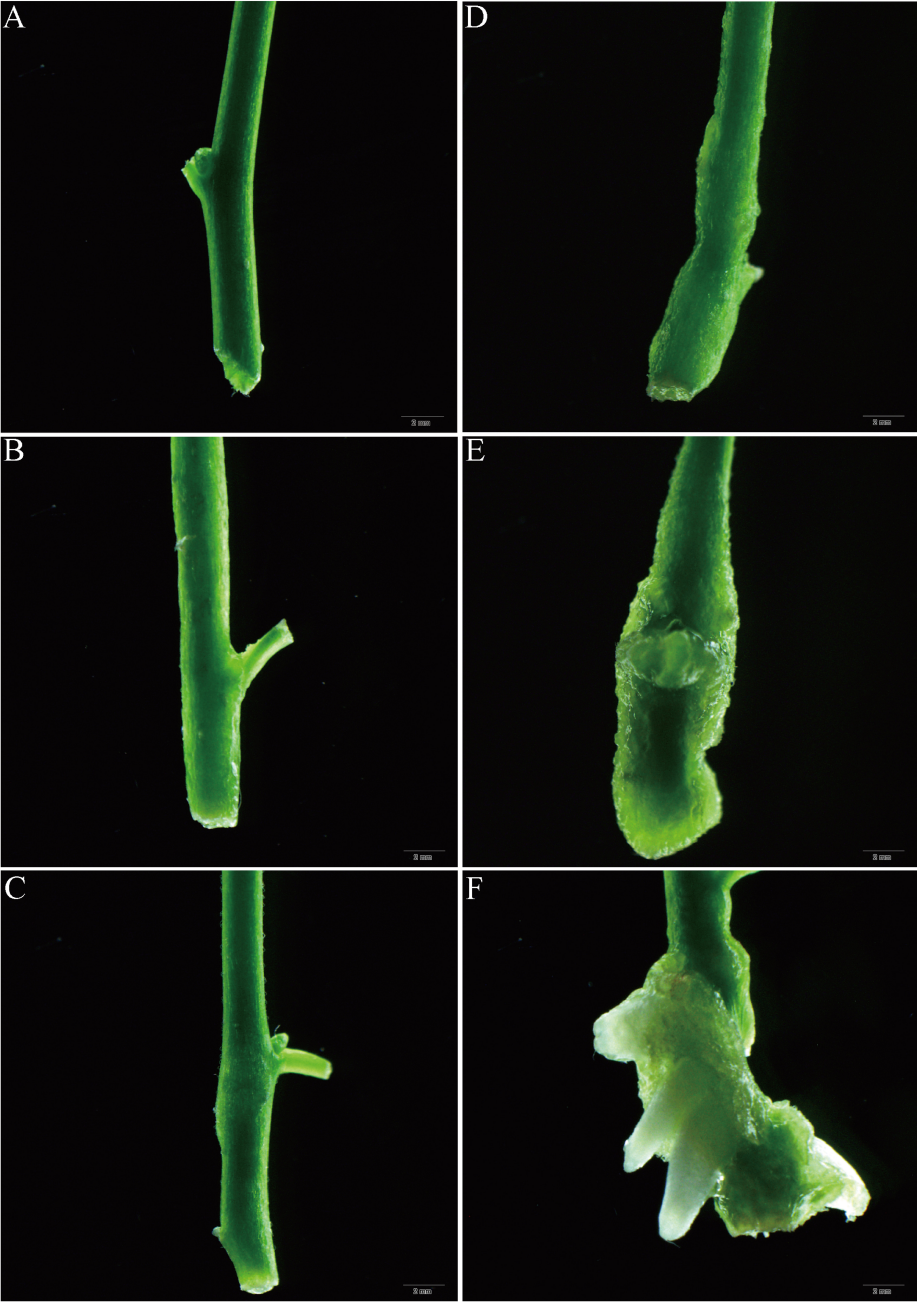
Different periods of 7dN3 group on AR induction and growth morphology under stereomicroscope. **(A-F)** corresponds to culture for 0, 3, 5, 7, 9, and 11 days respectively.

**Figure 6:**
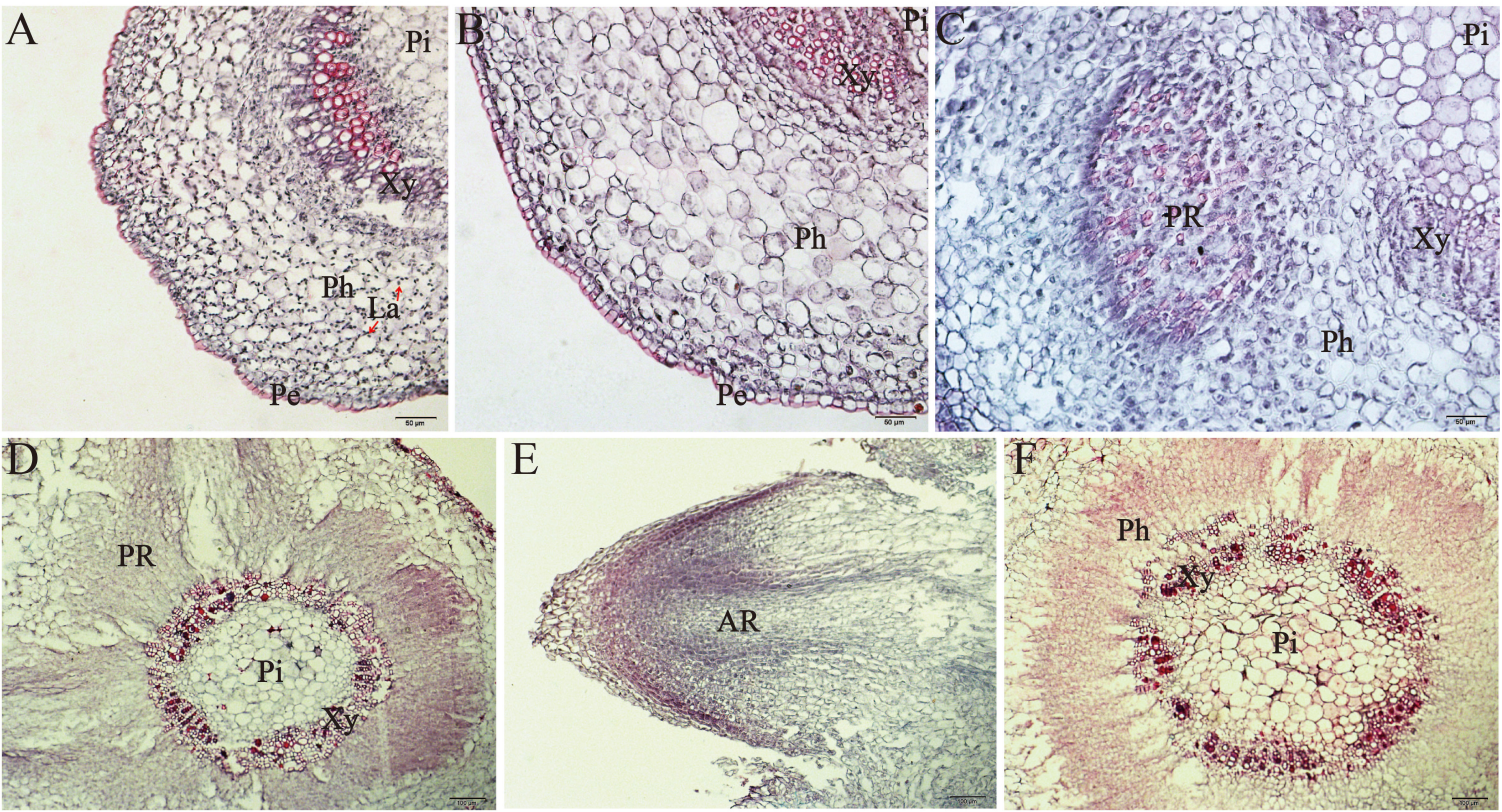
Anatomical observations of AR formation in stem cuttings of *E. ulmoides*treated by 7dN3 group and N3 group. **(A)** 7dN3 group in 0 d. **(B)** 7dN3 group in 5 d. **(C)** 7dN3 group in 7 d. **(D, E)** 7dN3 group in 11 d. **(F)** N3 group in 11 d. Pe, Periderm; Ph, Phloem; Xy, Xylem; Pi, Pith; La, Laticifer cell; AR, Adventitious root; PR, Root primordium.

Under stereomicroscopic observation (**Figures 5A–F**), we observed the swelling of the stem base of the shoots of the 7dN3 treatment starting on the third day (**Figure 5B**). This swelling subsequently progressed, leading to the formation of callus tissue (**Figures 5B–E**). Furthermore, the emergence of adventitious roots was noted to occur on the eleventh day (**Figure 5F**). Anatomical observations indicate that with increasing NAA treatment time (**Figures 6A–C**), the cytoplasm of cells in the cambium area and adjacent phloem became more densely stained and protruded towards the cortical tissue (**Figure 6C**). This active cell division resulted in the outline formation of the root primordium. The AR primordium cells continue to divide, resulting in an expansion of the quantity and size of root cells. Simultaneously, the root tip begins to differentiate and extend into the cortex. As the AR primordia continues to elongate, on day 11, it finally pierces the stem’s cortex and epidermis (**Figures 5F**, **6D, E**). Nonetheless, up until day 11, there were no indications that AR was forming in the control samples (**Figure 6F**). Anatomical data demonstrates the formation of distinct meristems on day 7 (**Figure 6C**). During this period, a variety of cytological events occurred, from the initial cell division to the formation of meristems. It is also the optimal duration for auxin treatment to yield the highest root induction rate. On day 7, the root meristem is established, rendering root primordium development auxin-independent thereafter. Consequently, after the 7th day, the application of auxin must be discontinued. The rooting process of apple rootstocks yielded similar results (Naija et al., 2008).

## Conclusion

Here, we propose an efficient regeneration system for *E. ulmoides* based on the direct organogenesis pathway of axillary buds in microstem segments. The above-mentioned procedure has the shoots of high sprouting efficiency, high reproduction coefficient, and a low coefficient of variation. When the explants were grown on the DKW medium with 2.0 mg L^−1^ tZ, the maximum shoot sprouting frequency (100%) and the best shoot elongation effects were achieved. In addition, the highest average bud elongation height (5.18 cm) was observed in cultured elongated shoots in DKW medium supplemented with 2.0 mg L^−1^ tZ and 20.0 g L^−1^ sucrose. Furthermore, this study also introduces a novel two-step rooting method for *E. ulmoides*, which solve the challenges encountered in the rooting process and improved the efficiency of the regeneration system. This study serves as a foundation for the further utilization of *E. ulmoides* in various sectors including medicinal, healthy food and beverage, and EUR fields. However, the regulatory mechanisms of tZ on the sprouting and elongation of *E. ulmoides* shoots, and the mechanism of the inhibition of root growth by NAA in the two-step rooting method 7 days after application, and the interactions between hormones, remain poorly understood. These issues necessitate further molecular-level research to elucidate their underlying scientific principles. This study plays a crucial role in improving the genetic quality of elite *E. ulmoides* plants. Additionally, it can serve as a valuable resource for successfully propagating other woody plants that are known for their challenging root formation.

## Data Availability

The original contributions presented in the study are included in the article/**Supplementary Material**. Further inquiries can be directed to the corresponding author/s.
