# Supplementary material for: Efficient plant regeneration through direct shoot organogenesis and two-step rooting in Eucommia ulmoides Oliver
Source: Front Plant Sci. 2024 Sep 20;15:1444878. doi: 10.3389/fpls.2024.1444878 (PMC11449753; doi:10.3389/fpls.2024.1444878)
Supplement: Supplementary file 1 [file Table1.docx]

**Supplementary material**


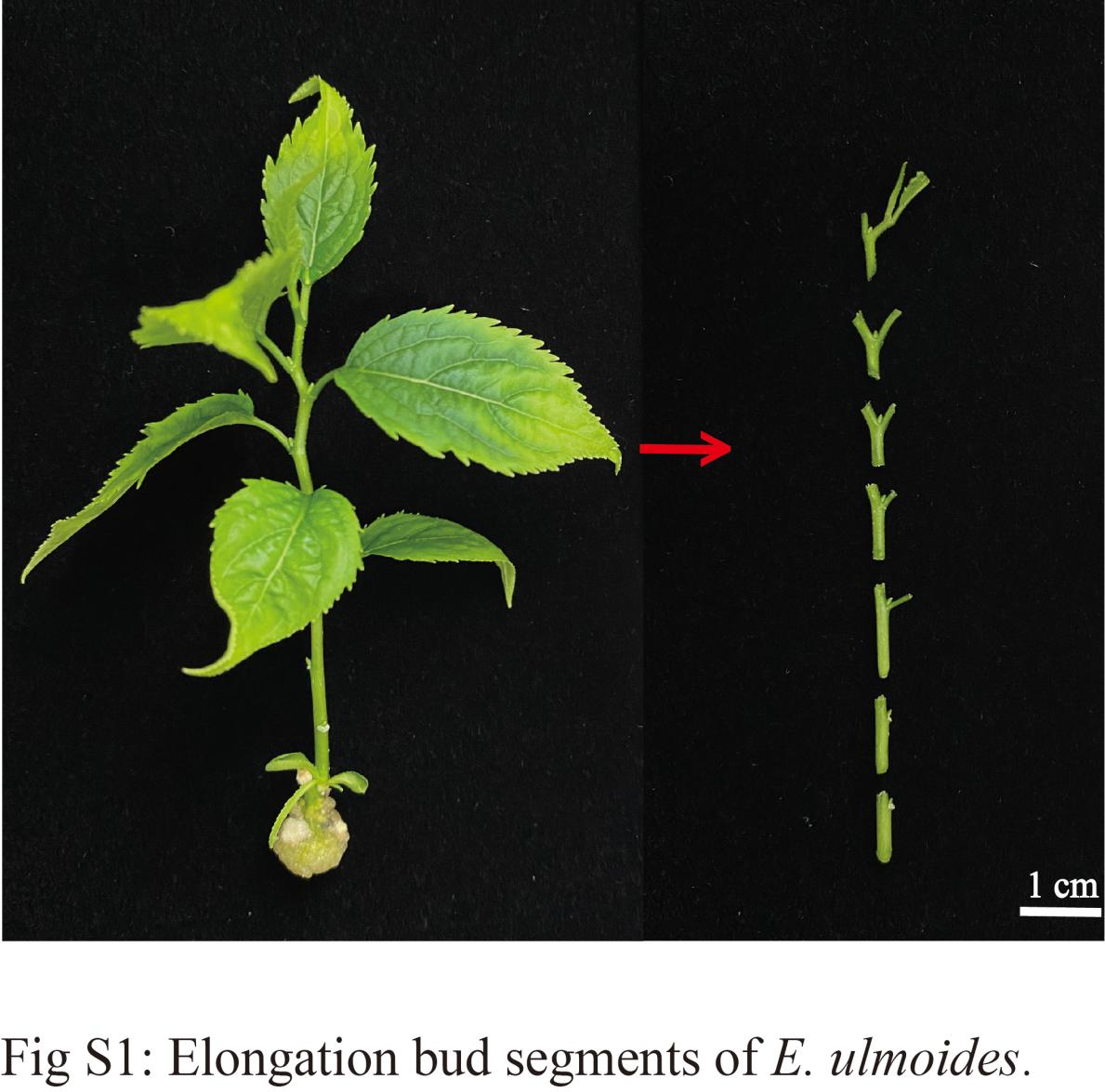


**Supplementary Fig 1**: Elongation bud segments of *E. ulmoides.*

**Supplementary Fig 2**: Rooting process in three experimental groups of *E. ulmoides.*

**
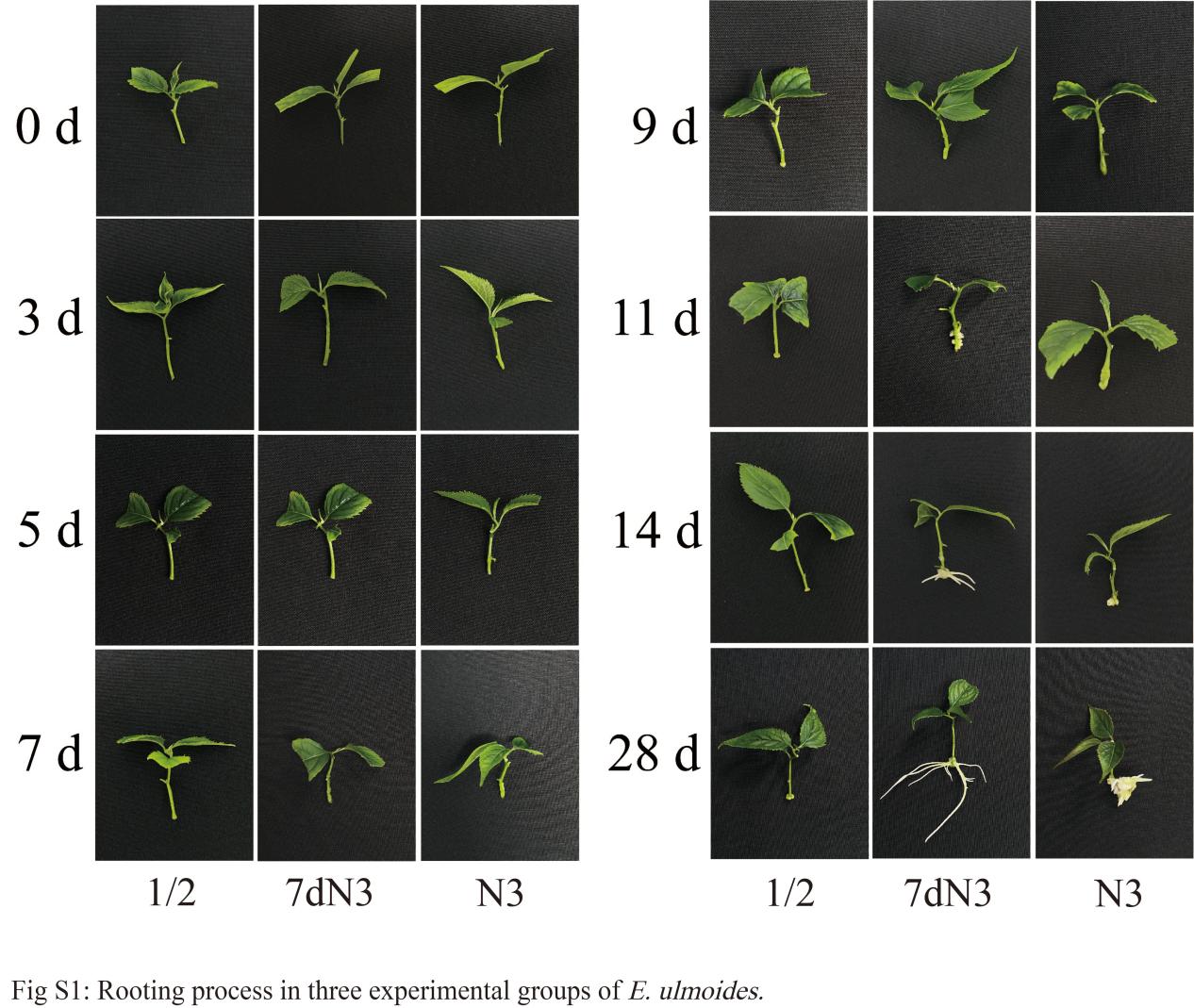
**
